# Supplementary material for: Somatic Mobilization: High Somatic Insertion Rate of mariner Transposable Element in Drosophila simulans
Source: Insects. 2022 May 12;13(5):454. doi: 10.3390/insects13050454 (PMC9144738; doi:10.3390/insects13050454)
Supplement: Supplementary file 1 [file insects-13-00454-s001.zip › insects-1710231-supplementary.pdf]

Supplementary Material

Somatic Mobilization: high somatic insertion rate of mariner transposable element in Drosophila simulans

Mariana Cancian, Tiago Minuzzi Freire da Fontoura Gomes and Elgion Lucio Silva Loreto

Supplementary Material S1 *(TISseq protocols)*

TISseq molecular protocol

DNA extraction

For the DNA extractions, 30 adults' female Drosophila were used at 20ºC and 28ºC, containing from 1 to 4 days. For extraction, DNA purification kit (Ludwig Biotecnologia LTDA) was used.

Cleavage with restriction enzymes

The total DNA was cleaved by two restriction enzymes: HindIII and EcoRI. We used 1ug of DNA, 5U of EcoRI, 5U of HindIII, RE 1x buffer E, 2ug of Acetylated BSA, and Milli-Q water treated with DEPC (diethylpyrocarbonate) were used to complete the final volume of 15uL. The samples were left for 4 hours at 37ºC and the enzymes were inactivated at 65ºC for 15 minutes.

Adapter annealing and phosphorylation

Three oligonucleotides were synthesized for the construction of the two cohesive adapters. Both for the upstream and downstream ends to the *mariner,* we have the following adapters: eco_cohesive for EcoRI and hind_cohesive for HindIII (table 1). The two adapters have the same sequence, changing only the first four nucleotides corresponding to the sequence of the restriction enzymes. As a complement to both strands, the compl_adapt adapter was made (table 1).

Supplementary Table S1. *Adapters and primer sequences.*

**adapters sequences**	
eco_cohesive	5' AATTAGATCGGAAGAGCGTCGTGTAGGGAAAGAGTG 3'
hind_cohesive	5' AGCTAGATCGGAAGAGCGTCGTGTAGGGAAAGAGTG 3'
compl_adapt	5' CACTCTTTCCCTACACGACGCTCTTCCGATCT 3'
**primer sequences**	
Mos5_F	5' CACTCTTTCCCTACACGACGCTCTTCCGATCT 3'

Mos5_R[1]	5' **GTGACTGGAGTTCAGACGTGTGCTCTTCCGATCT**CGAACCGACATTCCCTACTT 3'
Mos3_F	5' CACTCTTTCCCTACACGACGCTCTTCCGATCT 3'
Mos3_R[1]	5' **GTGACTGGAGTTCAGACGTGTGCTCTTCCGATCT**GCGACGGCAAATACTTAGAATAA 3'

[1] Bold strings are Illumina adapters for sequencing.

The oligonucleotides were annealed to make double-stranded adapters. 100pmol of eco-cohesive oligonucleotide, 100pmol of compl_adapt, 1x PCR buffer were used and milliq water treated with DEPC were added to complete 20uL. The same was done for the hind_cohesive oligo. The two tubes were placed at 65ºC for 5 minutes and were allowed to cool to room temperature, gradually.

Therefore, the annealed adapters were phosphorylated by the enzyme T4 Polynucleotide Kinase to favor binding to DNA. 1x Reaction Buffer Kinase, 7-15U of T4 Polynucleotide Kinase, 1mM of 10mM ATP were used and added with water to a final volume of 40uL were added. The samples were kept at 37ºC for 30 minutes. The enzyme was inactivated at 68ºC for 10 minutes.

Adapter's connection to cleaved DNA

To 1ug of cleaved DNA, 2.5uM of each eco and hind cohesive adapters were added. For annealing, the samples were placed at 65ºC for 5 minutes and then cooled gradually to room temperature (approximately 0.5ºC / min). After annealing adapters and cleaved DNA, the samples remained at 4ºC for 1 hour and afterwards 1x Ligase Reaction Buffer, 4U T4 DNA Ligase and 1mM ATP were added. The samples remained overnight at 4°C. The reaction was inactivated at 65ºC for 20 minutes.

Primers and amplification

The primer pair Mos_5 (Table 1) and Compl_adapt (table 1) amplifies an upstream fragment to the *mariner*, while the primer pair Mos_3 (Table 1) and Compl_adapt, amplifies a downstream fragment to the *mariner*. For amplification, 8ng of DNA cleaved with adapters, 1x PCR Buffer, 10mM dNTP, 10 μM of each primer, 50mM MgCl2, 1U of Taq DNA Polymerase (Invitrogen) and milli-Q water treated with DEPC were used. PCR conditions were 95°C for 5 minutes (Polymerase Activation), followed by 40 cycles of 30 seconds at 95°C, 30 seconds at 60°C and 2 minutes at 72°C (PCR Cycling), followed by 10 minutes at 72°C and 4°C indefinitely. After each PCR, another PCR was performed using samples of the first as a template, in order to increase the number of fragments of interest. The conditions of the second PCR were identical to the initial PCR, using 5uL of the initial PCR as a template for the second.

Supplementary Material S2

TISseq-Pipe Pipeline description

Some external files were used during the pipeline. The sequences of the primer pairs used for the amplifications (referring to the *mariner*, both at the upstream and downstream ends Table 1). These sequences of the primers that annealed the *mariner* are used as query sequence, separating the interest reads and discarding sequencing chimeras and amplified fragments that do not contain *mariner*. In addition, a file containing the adapter sequence that connected to the restriction enzyme site was used. The module 1 can be substituted for a default trimming, with an automatic trimmer (ex, TrimGalore). However, we strongly recommend that specific trimming is performed to eliminate every sequence of artefacts as possible. This module is important to evit false positives in the Blast analysis (figure supplementary 1).

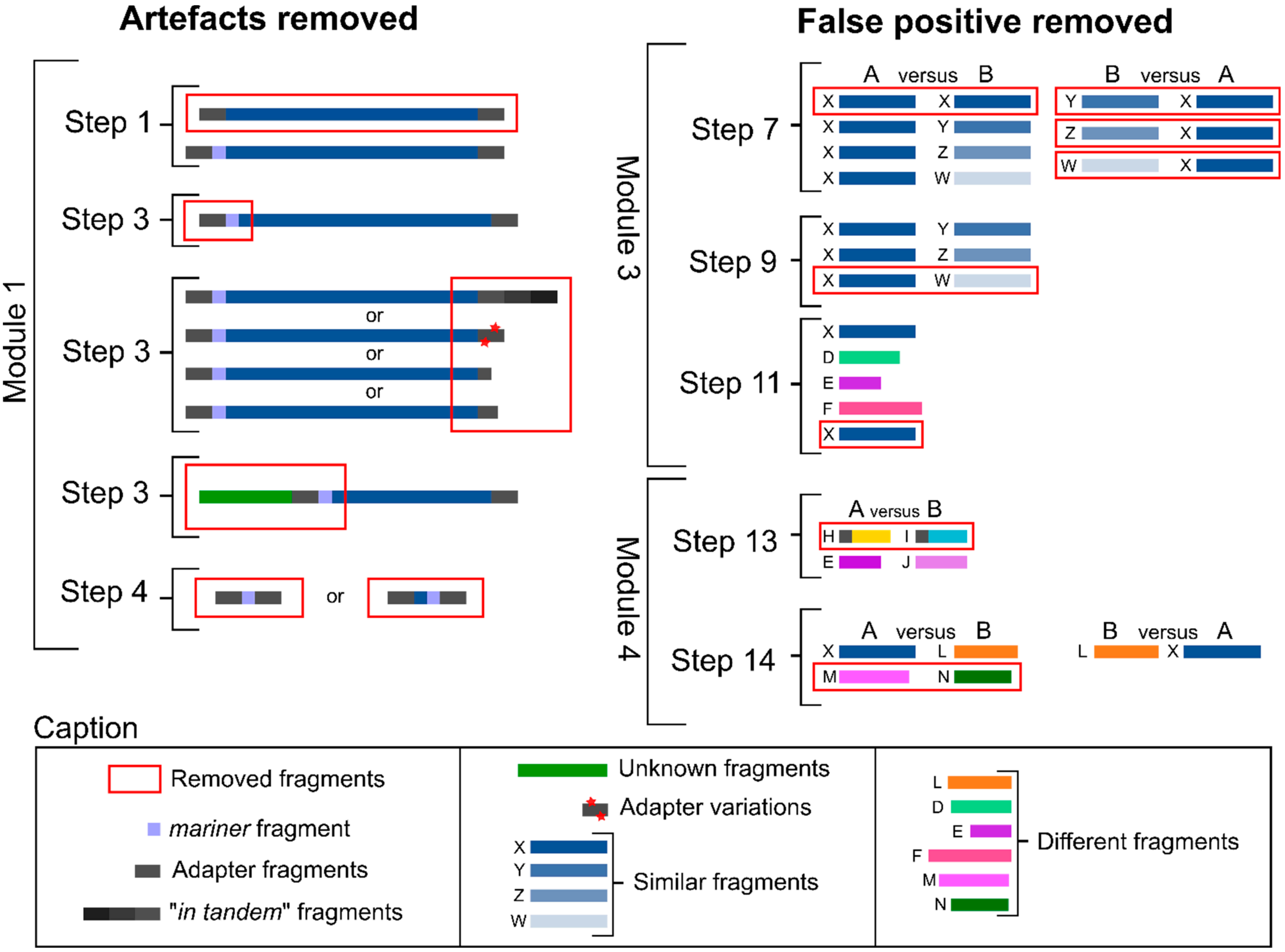

Figure supplementary S1. Sequencing artifacts and false positives fragments. In Module 1 are represented all identified artifacts fragments in the samples and steps that were removed. In Modules 3 and 4 are represented all false positives fragments found in the samples and steps that they were removed.

Module 1: Trimming

Step 1- *mariner* separation: Selection of fragments with *mariner* at one end. The input file and the output file have the extension fastq.

Step 2- Output modification: The fastq_to_fasta program - within the fastx tool kit, version 0.0.14 - was used to convert the files.

Step 3- Trimming: two trimming were performed - one for each end of the fragment of interest. At the 5' end of the fragment of interest, different chimeras of the adapter linked to the restriction enzyme site were observed. To define the variations of the adapters found, an alignment was performed - using blast - of the known sequence of the adapter with all files, before any trimming. The sequences of all the chimeras found in all the samples were concatenated in a file and the repetitions were removed. It found 30 adapter variations at the 5' end and removed the concatenate from all sample files. At the 3' end of the fragment, the sequence refers to the primer and everything after it was removed - adapter plus sequencing artifacts.

Step 4- Size threshold: A minimum size threshold of 25 nucleotides were defined for further analysis.

Módulo 2: Selfblast and output organisation

Step 5- Selfblast: Among the fragments of interest, there are fragments with and without coverage.To define these fragments, blast from each of the 12 samples was used using the same sample as query and subject. In this step, the input file has a fasta extension and tsv output (Tab Separated Values).

Step 6- Coverage separation: In the selfblast, there are sequences in which alignment presents only one hit (single sequences or without coverage) and sequences that present multiple hits (multiple sequences or with coverage). Each sample was separated into two files, one with coverage and other without it for further analysis.

Module 3: Removal of redundant sequences

Step 7- Filter 1: consists of removing redundancies from alignment. In sequences with coverage, the blasts of the sequence against itself were removed. In addition, blast redundancies, such as A versus B and, subsequently, B versus A, were excluded. Only one of the hits was maintained.

Step 8- Scov creation: To define whether the sequences compared were the same or not, two parameters were defined: qCOV (query coverage) and sCOV (subject coverage). The value of qCOV is calculated from the ratio between the aligned size and the size of the query string, multiplied by 100. The sCOV is calculated in the same way, considering the subject string.

Step 9- Definition of the representative fragment between the repetitions: A threshold of values was defined to determine whether the sequences were from the same site in the genome or from a different site. It was defined that sequences that had qCOV and/or sCOV greater than or equal to 70 would be considered sequences from the same location and with lower values, would be different sequences. For the sequences considered from the same location, a representative fragment was selected. Meanwhile, the slightly similar sequences, with q/sCOV <70, were disregarded.

Step 10- Get sequence: From the IDs defined as representatives, the sequences referring to them were retrieved. In this step, the input file with the IDs of each sample was used and searched as a query sequence in the fasta files of step 4 referring to each sample.

Step 11- Filter 2: consists of removing the repeated sequences that are still left in the samples. Filter additions serve to ensure that the next steps happen with the highest sensitivity and with the lowest occurrence of false positives.

Module 4: Concatenated sequences

Step 12- Get sequence: in the same way as performed for the sequences with coverage in step 10, the without coverage sequences were retrieved by the IDs generated in step 6 and retrieved from the files of step 4.

Step 13- Concatenate the fragments with and without coverage: The two fasta sequences were concatenated in a single file of each sample, joining with and without coverage sequences. This is because without coverage sequences do not need to go through the other steps in the pipeline. After this joining, the steps of module 5 were followed.

Module 5: Sample and Reciprocal Blast

Step 14- Samples Blast (Alignment of one sample against another): A comparison was made between the following: 20ºC versus 28ºC and vice versa; Each comparison was carried out at the two ends of the element, upstream end against itself, and in the same way at the downstream end of the *mariner*.

Step 15- Filter 3: Despite the trimming, alignments of adapter variations between the samples were still seen. The Blast output files were trimmed in order to remove possible false positive alignments due to these sequences.

Step 16- RBH (Reciprocal Blast): Reciprocal blast was performed in order to give greater specificity and sensitivity between the alignments. To define the values of the results there were used: the FASTA files from step 13 as the total number of positions; the result of the reciprocal blast as equal positions in the two samples; and, it was performed a subtraction of the two values to find the positions that are unique in these samples (0 hits).

Supplementary Table S2. *Raw data and detailed results of Pipeline.*

	**Total reads**	**Upstream and downstream reads**		**>25nt**	**TISseq with coverage**	**TISseq without coverage**
**20ºC A**	6846 reads	up	2798 reads	2022 reads	134 fragments	1529 fragments
		down	2173 reads	1610 reads	103 fragments	1205 fragments
**20ºC B**	11253 reads	up	5370 reads	3708 reads	357 fragments	2500 fragments
		down	3442 reads	3022 reads	295 fragments	2157 fragments

**20ºC C**	14055 reads	up	6463 reads	4869 reads	499 fragments	3455 fragments
		down	4366 reads	3968 reads	382 fragments	2979 fragments
**28ºC A**	7524 reads	up	2840 reads	2418 reads	161 fragments	1945 fragments
		down	2262 reads	2032 reads	111 fragments	1651 fragments
**28ºC B**	10836 reads	up	4764 reads	3990 reads	438 fragments	2952 fragments
		down	3485 reads	2978 reads	229 fragments	2338 fragments
**28ºC C**	13747 reads	up	5002 reads	3057 reads	462 fragments	1878 fragments
		down	3558 reads	893 reads	102 fragments	644 fragments

Supplementary Table S3. Detailed data of somatic insertion rate.

**ID**	**Fragments trimmed**	**Fragments without coverage**	**Fragments with coverage**	**Total fragments**	**Insertion per genome**	**Insertion estimative in 100% of genome**	**Ratio**
**20ºC A upstream**	2022	1395	134	1529	50,9	171,03	0,08
**20ºC B Upstream**	3708	2143	357	2500	83,33	279,6	0,07
**20ºC C upstream**	4869	2956	499	3455	115,16	386,5	0,08
**20ºC upstream**				2494,6 (+-963,01)	83,1 (+-32,1)	279,04 (+-107,7)	0,08 (+- 0,005)
**28ºC A Upstream**	2418	1784	161	1945	64,83	217,6	0,09
**28ºC B upstream**	3990	2514	438	2952	98,4	330,2	0,08

**28ºC C upstream**	3057	1416	462	1878	62,6	210,1	0,07
**28ºC upstream**				2258,3 (+-601,6)	75,3 (+-20,05)	252,6 (67,3)	0,08 (+-0,01)
**20ºC A Downstream**	1610	1102	103	1205	40,16	134,8	0,08
**20ºC B Downstream**	3022	1862	295	2157	71,9	241,3	0,08
**20ºC C Downstream**	3968	2597	382	2979	99,3	333,2	0,08
**20ºC Downstream**				2113,6 (+-887,8)	70,4 (+-29,6)	236,4 (+-99,3)	0,08 (+-0,002)
**28ºC A Downstream**	2032	1540	111	1651	55,03	184,7	0,09
**28ºC B Downstream**	2978	2109	229	2338	77,93	261,5	0,09
**28ºC C Downstream**	893	542	102	644	21,46	72,03	0,08
**28ºC Downstream**				1544,3 (+-852,02)	51,5 (+-28,4)	172,7 (+-95,3)	0,09 (+-0,005)

Supplementary Table S4. Detailed data of Blast alignment sequences.

mariner up								
id	Description	Max Score	Total Score	Query Cover	E value	Per. ident	Acc. Len	Accession
63c37770-a066-4e81-8fa2-176bf1b322dd	*Drosophila melanogaster* chromosome 2L	2588	60457	100%	0	90	23513712	AE014134.6
	Drosophila simulans H4, H2A, H2B, H1 genes for histone 4, histone 2A, histone 2B, histone 1, complete cds	2584	2584	82%	0	94,94	4635	AB055959.1
ccee4b35-af37-446c-a5c7-4cf248f20893	*Drosophila melanogaster* chromosome 2L	2697	62996	100%	0	90,96	23513712	AE014134.6
	Drosophila simulans H4, H2A, H2B, H1 genes for histone 4, histone 2A, histone 2B, histone 1, complete cds	2684	2684	82%	0	95,94	4635	AB055959.1
6c8cbd9f-6f52-4910-b3f9-0ceb7e7271de	*Drosophila melanogaster* chromosome 2L	2719	63500	100%	0	91,13	23513712	AE014134.6
	Drosophila simulans H4, H2A, H2B, H1 genes for histone 4, histone 2A, histone 2B, histone 1, complete cds	2699	2699	82%	0	96,11	4635	AB055959.1
cdc765e8-b3c0-484a-94d8-06017d6855bc	*Drosophila melanogaster* chromosome 2L	2760	64447	99%	0	91,59	23513712	AE014134.6
	Drosophila simulans H4, H2A, H2B, H1 genes for histone 4, histone 2A, histone 2B, histone 1, complete cds	2813	2813	82%	0	97,25	4635	AB055959.1
6a57e642-	*Drosophila melanogaster* chromosome 2L	2549	59435	100%	0	89,62	23513712	AE014134.6

f4b1-49ff-9b5a-2b3d85fb4872	*Drosophila simulans* H4, H2A, H2B, H1 genes for histone 4, histone 2A, histone 2B, histone 1, complete cds	2481	2481	81%	0	93,94	4635	AB055959.1
d742d884-ef7f-4b78-b7f2-6861ef9e1ad1	*Drosophila melanogaster* chromosome 2L	2577	60314	100%	0	89,92	23513712	AE014134.6
	Drosophila simulans H4, H2A, H2B, H1 genes for histone 4, histone 2A, histone 2B, histone 1, complete cds	2580	2580	82%	0	94,89	4635	AB055959.1
0c76dbaf-6035-4c02-ae30-46ed7fc0b44e	*Drosophila melanogaster* chromosome 2L	2409	56166	100%	0	88,43	23513712	AE014134.6
	Drosophila simulans H4, H2A, H2B, H1 genes for histone 4, histone 2A, histone 2B, histone 1, complete cds	2379	2379	81%	0	92,81	4635	AB055959.1
5107b2c8-5e39-49fb-baf8-e0371e5886ab	*Drosophila melanogaster* chromosome 2L	2396	55905	100%	0	88,43	23513712	AE014134.6
	Drosophila simulans H4, H2A, H2B, H1 genes for histone 4, histone 2A, histone 2B, histone 1, complete cds	2405	2405	80%	0	93,54	4635	AB055959.1
9b45f66d-1c82-4a73-89ce-38eaf5b81dd0	*Drosophila melanogaster* chromosome 2L	2270	52857	100%	0	87,24	23513712	AE014134.6
	Drosophila simulans H4, H2A, H2B, H1 genes for histone 4, histone 2A, histone 2B, histone 1, complete cds	2340	2340	80%	0	92,96	4635	AB055959.1
246cae22-9829-4f5a-af95-	*Drosophila melanogaster* chromosome 2L	2436	56756	100%	0	88,88	23513712	AE014134.6
	Drosophila simulans H4, H2A, H2B, H1 genes for	2471	2471	83%	0	93,58	4635	AB055959.1

69ad56b3a3a4	histone 4, histone 2A, histone 2B, histone 1, complete cds							
b55c18f3-c209-4b94-914e-5446c8f43630	*Drosophila melanogaster* chromosome 2L	604	13896	62%	3E-170	84,35	23513712	AE014134.6
	Drosophila simulans H4, H2A, H2B, H1 genes for histone 4, histone 2A, histone 2B, histone 1, complete cds	745	745	94%	0	81,59	4635	AB055959.1
fc5cb8d7-685f-483a-a181-ee8ca6eb0513	*Drosophila melanogaster* chromosome 2L	1467	34145	99%	0	80,45	23513712	AE014134.6
	Drosophila simulans H4, H2A, H2B, H1 genes for histone 4, histone 2A, histone 2B, histone 1, complete cds	1539	1621	81%	0	84,44	4635	AB055959.1
3d5debe6-c94d-4681-959c-15033fdf9639	*Drosophila melanogaster* chromosome 2L	2567	59795	100%	0	89,86	23513712	AE014134.6
	Drosophila simulans H4, H2A, H2B, H1 genes for histone 4, histone 2A, histone 2B, histone 1, complete cds	2519	2519	82%	0	94,12	4635	AB055959.1
bef99909-6de0-4d1d-8be3-04c3296896f3	*Drosophila melanogaster* chromosome 2R	2891	2891	99%	0	92,92	25286936	AE013599.5
	PREDICTED: *Drosophila simulans* putative inorganic phosphate cotransporter (LOC6735767), mRNA	1764	2612	77%	0	95,99	1958	XM_002082644.4
6c678414-d51a-4422-876a-c90a255236bd	*Drosophila melanogaster* chromosome 3L	2586	2586	97%	0	90,59	28110227	AE014296.5

73c40db5-56a8-4349-a3e5-f10fef0e621d	*Drosophila melanogaster* chromosome 3L	2259	2259	97%	0	87,59	28110227	AE014296.5
00030b24-6d59-47d3-aca1-2aeb33788ec6	*Drosophila melanogaster* chromosome 3L	1864	2046	89%	0	88,77	28110227	AE014296.5
a4baedec-cd33-43aa-9750-ac54afc86983	*Drosophila simulans* white gene, complete cds	2154	2154	98%	0	86,69	5805	U64875.1
	Drosophila melanogaster chromosome X	1168	1611	93%	0	82,17	23542271	AE014298.5
c507810c-31b0-4ec8-9ba7-05439d7b51fa	*Drosophila simulans* white gene, complete cds	2436	2436	100%	0	88,71	5805	U64875.1
	Drosophila melanogaster chromosome X	1387	1831	97%	0	84,17	23542271	AE014298.5
a670f244-36eb-4296-9ff1-2de29cb02f18	*Drosophila simulans* white gene, complete cds	747	979	92%	0	77,86	5805	U64875.1
	Drosophila melanogaster chromosome X	438	539	65%	6E-120	75,54	23542271	AE014298.5
***mariner* down**								
**id**	**Description**	**Max Score**	**Total Score**	**Query Cover**	**E value**	**Per. ident**	**Acc. Len**	Accession
63c37770-	*Drosophila melanogaster* chromosome 2L	761	30796	94%	0	95,61	23513712	AE014134.6

a066-4e81-8fa2-176bf1b322dd	*Drosophila simulans* H4, H2A, H2B, H1 genes for histone 4, histone 2A, histone 2B, histone 1, complete cds	1037	1856	94%	0	97,24	4635	AB055959.1
ccee4b35-af37-446c-a5c7-4cf248f20893	PREDICTED: *Drosophila simulans* histone H2B-like (LOC120285809), misc_RNA	231	231	99%	6E-59	97,78	471	XR_005545073.2
	PREDICTED: *Drosophila simulans* histone H2B (LOC120285808), mRNA	231	231	99%	6E-59	97,78	491	XM_039298424.2
6c8cbd9f-6f52-4910-b3f9-0ceb7e7271de	*Drosophila melanogaster* chromosome 2L	1024	57765	99%	0	89,68	23513712	AE014134.6
	Drosophila simulans H4, H2A, H2B, H1 genes for histone 4, histone 2A, histone 2B, histone 1, complete cds	1190	3155	99%	0	96,2	4635	AB055959.1
cdc765e8-b3c0-484a-94d8-06017d6855bc	*Drosophila melanogaster* chromosome 2L	1072	60306	99%	0	90,64	23513712	AE014134.6
	Drosophila simulans H4, H2A, H2B, H1 genes for histone 4, histone 2A, histone 2B, histone 1, complete cds	1260	3279	99%	0	97,95	4635	AB055959.1
6a57e642-f4b1-49ff-9b5a-2b3d85fb4872	*Drosophila melanogaster* chromosome 2L	1055	55270	99%	0	89,92	23513712	AE014134.6
	Drosophila simulans H4, H2A, H2B, H1 genes for histone 4, histone 2A, histone 2B, histone 1, complete cds	1197	3015	99%	0	92,99	4635	AB055959.1
d742d884-ef7f-4b78-b7f2-6861ef9e1ad1	*Drosophila melanogaster* chromosome 2L	2398	56059	99%	0	88,69	23513712	AE014134.6
	Drosophila simulans H4, H2A, H2B, H1 genes for	3073	3073	100%	0	94,36	4635	AB055959.1

	histone 4, histone 2A, histone 2B, histone 1, complete cds							
0c76dbaf-6035-4c02-ae30-46ed7fc0b44e	*Drosophila melanogaster* chromosome 2L	1127	55606	99%	0	91,7	23513712	AE014134.6
	Drosophila simulans H4, H2A, H2B, H1 genes for histone 4, histone 2A, histone 2B, histone 1, complete cds	1286	3050	99%	0	95,07	4635	AB055959.1
5107b2c8-5e39-49fb-baf8-e0371e5886ab	*Drosophila melanogaster* chromosome 2L	1611	55651	98%	0	88,82	23513712	AE014134.6
	Drosophila simulans H4, H2A, H2B, H1 genes for histone 4, histone 2A, histone 2B, histone 1, complete cds	2898	2898	99%	0	92,75	4635	AB055959.1
9b45f66d-1c82-4a73-89ce-38eaf5b81dd0	*Drosophila melanogaster* chromosome 2L	961	53494	99%	0	87,94	23513712	AE014134.6
	Drosophila simulans H4, H2A, H2B, H1 genes for histone 4, histone 2A, histone 2B, histone 1, complete cds	1136	2902	99%	0	94,99	4635	AB055959.1
246cae22-9829-4f5a-af95-69ad56b3a3a4	*Drosophila melanogaster* chromosome 2L	652	20314	92%	0	91,91	23513712	AE014134.6
	Drosophila simulans H4, H2A, H2B, H1 genes for histone 4, histone 2A, histone 2B, histone 1, complete cds	693	1056	92%	0	93,36	4635	AB055959.1
b55c18f3-c209-4b94-914e-5446c8f43630	*Drosophila melanogaster* chromosome 2L	994	31238	99%	0	79,58	23513712	AE014134.6
	Drosophila simulans H4, H2A, H2B, H1 genes for histone 4, histone 2A, histone 2B, histone 1, complete	1581	1970	99%	0	85,76	4635	AB055959.1

	cds							
fc5cb8d7-685f-483a-a181-ee8ca6eb0513	no hit							
3d5debe6-c94d-4681-959c-15033fdf9639	PREDICTED: *Drosophila simulans* histone H2B (LOC120285806), mRNA	665	665	86%	0	95,5	472	XM_039298422.2
bef99909-6de0-4d1d-8be3-04c3296896f3	*Drosophila melanogaster* chromosome 2R	1742	1742	96%	0	88,75	25286936	AE013599.5
6c678414-d51a-4422-876a-c90a255236bd	no hit							
73c40db5-56a8-4349-a3e5-f10fef0e621d	*Drosophila melanogaster* chromosome 3L	885	1010	95%	0	80,49	28110227	AE014296.5
00030b24-6d59-47d3-aca1-2aeb33788ec6	*Drosophila melanogaster* chromosome 3L	1602	1602	100%	0	81,59	28110227	AE014296.5
a4baedec-cd33-43aa-9750-ac54afc86983	*Drosophila melanogaster* white locus	1310	1621	83%	0	83,5	14247	X02974.2
	Drosophila melanogaster chromosome X	1306	3400	98%	0	83,44	23542271	AE014298.5

c507810c-31b0-4ec8-9ba7-05439d7b51fa	*Drosophila melanogaster* chromosome X	628	960	90%	1E-177	88,2	23542271	AE014298.5
	Drosophila melanogaster white locus	627	964	91%	5E-177	88,2	14247	X02974.2
a670f244-36eb-4296-9ff1-2de29cb02f18	*Drosophila melanogaster* white locus	507	507	71%	2E-140	74,58	14247	X02974.2
	Drosophila melanogaster chromosome X	486	1313	87%	2E-134	74,37	23542271	AE014298.5

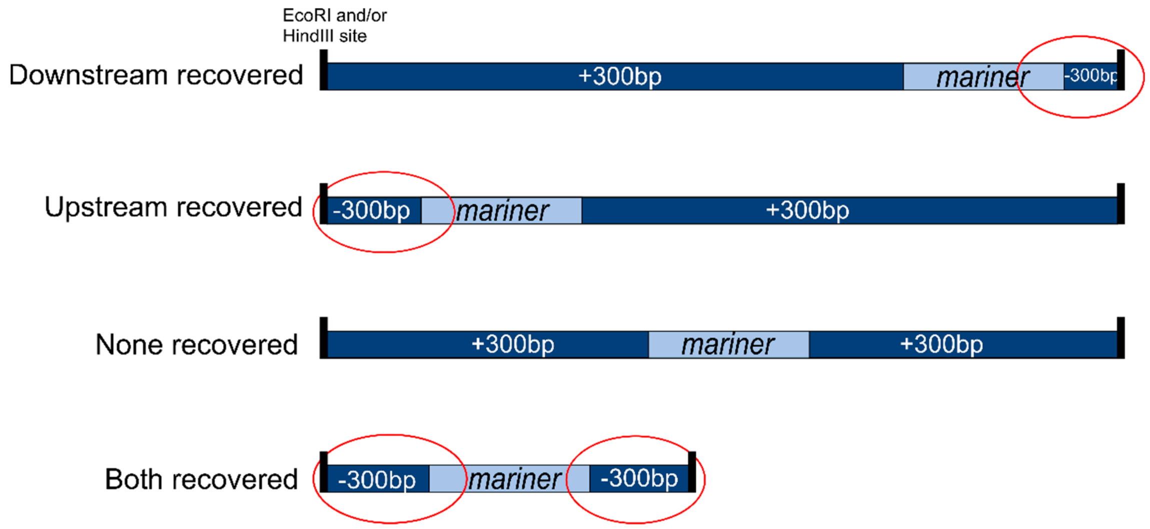

Figure supplementary S2. *mariner* ends sequenced. Depending if and where *mariner* is inserted, the end around element can be sequenced. If there is a site of restriction enzyme (EcoRI or HindIII) less and/or that 300bp of *mariner*, the fragment can be sequenced. Each element can have one end sequenced (only upstream or downstream), both ends, or nothing.

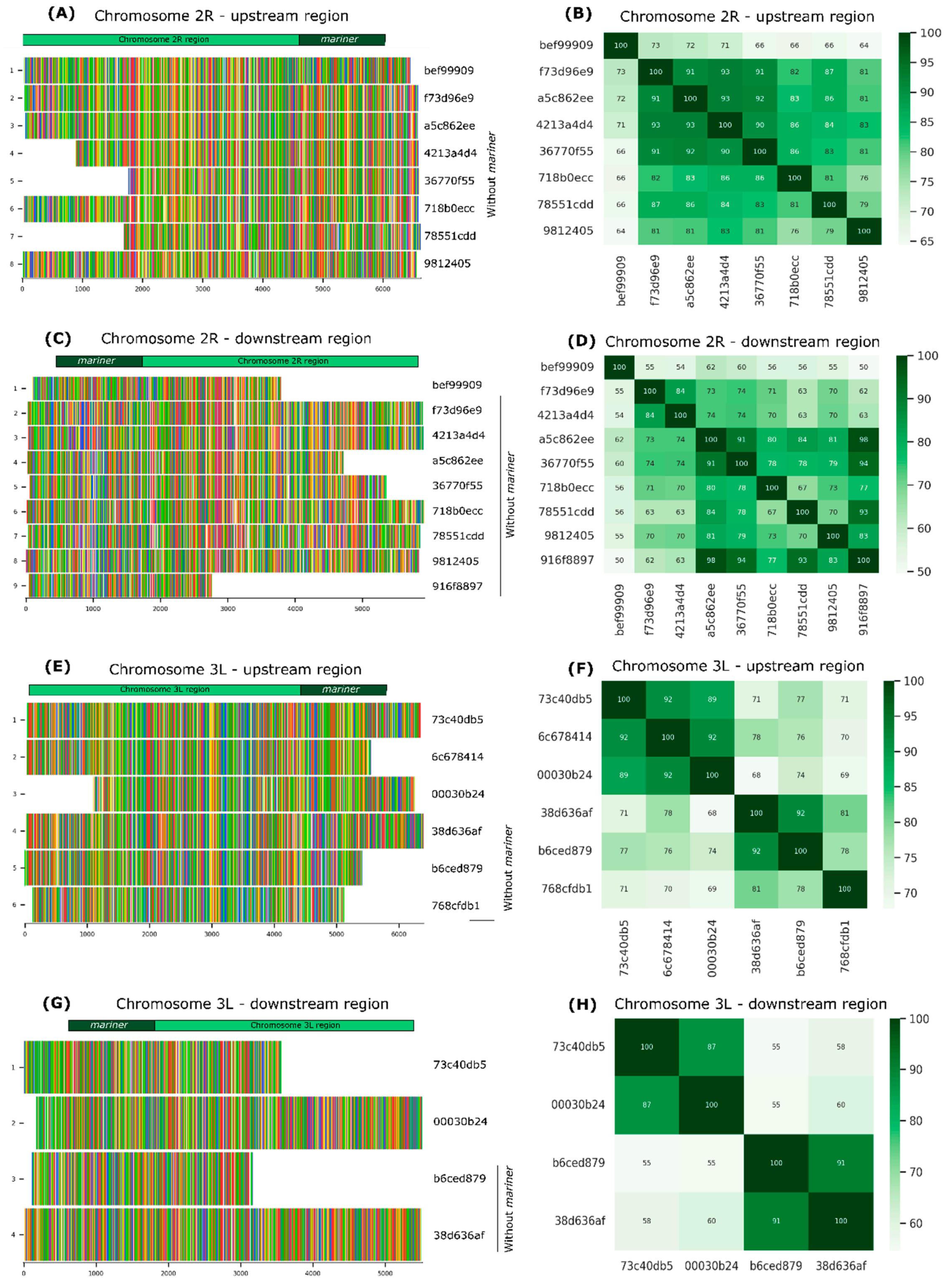

Figure supplementary S3. Alignment and heatmaps of regions upstream and downstream of *mariner*. (A) 8 fragments of upstream region of chromosome 2R, where one fragment contains *mariner* and 7 others do not; (B) Heatmap indicating the similarity between the 7 fragments with *mariner* differing from without; (C) and (D) represent the same thing of figures A and B, respectively, but in the region downstream of *mariner*; (E) Upstream region of chromosome 3L, representing the alignment of six sequences, 3 containing *mariner* e 3 without it; (F) Heatmap showing the similarity between the two groups of sequences: with *mariner* and without *mariner*; (G) Downstream region of chromosome 3L, but only with four sequences: two with mariner, and the other without; (H) Heatmap of downstream region of chromosome 3L, showing the two groups of similarity of sequences.
